# Genome Sequence of *Listeria monocytogenes* Strain F4244, a 4b Serotype

**DOI:** 10.1128/genomeA.01324-17

**Published:** 2017-12-07

**Authors:** Taylor W. Bailey, Naila C. do Nascimento, Arun K. Bhunia

**Affiliations:** a Department of Food Science, Molecular Food Microbiology Laboratory, Purdue University, West Lafayette, Indiana, USA; b Department of Comparative Pathobiology, Purdue University, West Lafayette, Indiana, USA

## Abstract

*Listeria monocytogenes* is an opportunistic invasive foodborne pathogen. Here, we performed whole-genome sequencing of *L. monocytogenes* strain F4244 (serotype 4b) using Illumina sequencing. The sequence showed 94.5% identity with strain F2365, serotype 4b, and 90.6% with EGD-e, serotype 1/2a.

## GENOME ANNOUNCEMENT

*Listeria monocytogenes* is a Gram-positive facultative anaerobic bacterial pathogen. Although presently ubiquitous in the environment, *L. monocytogenes* is often associated with foods such as raw produce, ready-to-eat deli meats, and unpasteurized dairy products. Listeriosis, or systemic infection with *L. monocytogenes*, is a potentially fatal condition, particularly in the immunocompromised population with a disproportionate risk of perinatal death and maternal infection in late-stage pregnancy. Given the high morbidity, albeit low incidence, *L. monocytogenes* is considered one of the most important microbial concerns within the food industry (1). The canonical infectious mechanism of *L. monocytogenes* is well described (2). However, work from our group has identified an alternative paracellular pathogenic pathway during the intestinal phase of infection and a discrepancy of this pathogenic phenotype across different *L. monocytogenes* strains influenced by the gene product of locus *lmo1634*, an AdhE homolog called the *Listeria* adhesion protein (3–7). The genome of *L. monocytogenes* strain F4244, serotype 4b, isolated from cerebrospinal fluid of a patient and obtained from the Centers for Disease Control and Prevention (CDC, Atlanta, GA) was sequenced to help elucidate a context for the differences observed for paracellular translocation.

The whole genome was sequenced from paired-end libraries (TruSeq DNA sample preparation kit, Illumina, San Diego, CA, USA) using 10% of an Illumina HiSeq 2500 lane. Reads were assembled using ABySS-PE v1.2.7 utilizing the reads with kmer set to 90 bases. Remaining gaps were closed using conventional PCR followed by Sanger sequencing giving a contiguous sequence of 2,994,740 bases. The resulting genome was annotated using the NCBI Prokaryotic Genome Annotation Pipeline (https://www.ncbi.nlm.nih.gov/genome/annotation_prok/). Annotation revealed 3,062 genes in total, of which 2,954 are protein coding, 85 are RNAs (14 rRNAs, 67 tRNAs, and 4 noncoding RNAs [ncRNAs]), and 23 are pseudogenes. Alignment of the determined genome with published genomes for *L. monocytogenes* Clip80459 (GenBank accession number NC_012488) and F2365 (NC_002973) show 94.8% and 94.5% identity, suggesting general conservation with the serotype 4b group of lineage I (8). This contrasts to *L. monocytogenes* strains EGD-e (NC_003210), EGD (NC_022568), and 10403S (NC_017544), with 90.6%, 89.9%, and 90.0% identities, respectively, all of serotype 1/2a group of lineage II (9). Analysis of the *lmo1634* locus (AdhE or LAP) from F4244 with these genomes demonstrates 99% sequence identity with Clip80459 and F2365 and 97% with EGD-e and EGD. This corresponds to a two-amino-acid substitution in Clip80459 and F2365 (G495V and S496Q), two additional substitutions in EGD and 10403S (K279N and A515S), and a fifth substitution in EGD-e (V202L).

### Accession number(s).

These whole-genome and plasmid sequences have been deposited at GenBank under the accession numbers CP015508 and CP015509, respectively.

## References

[1] Vázquez-Boland JA, Kuhn M, Berche P, Chakraborty T, Domínguez-Bernal G, Goebel W, González-Zorn B, Wehland J, Kreft J 2001 *Listeria* pathogenesis and molecular virulence determinants. Clin Microbiol Rev 14:584–640. doi:10.1128/CMR.14.3.584-640.2001. 11432815 PMC88991

[2] Cossart P 2011 Illuminating the landscape of host-pathogen interactions with the bacterium *Listeria monocytogenes*. Proc Natl Acad Sci U S A 108:19484–19491. doi:10.1073/pnas.1112371108. 22114192 PMC3241796

[3] Jagadeesan B, Koo OK, Kim KP, Burkholder KM, Mishra KK, Aroonnual A, Bhunia AK 2010 LAP, an alcohol acetaldehyde dehydrogenase enzyme in *listeria* promotes bacterial adhesion to enterocyte-like Caco-2 cells only in pathogenic species. Microbiology 156:2782–2795. doi:10.1099/mic.0.036509-0. 20507888

[4] Burkholder KM, Bhunia AK 2010 *Listeria monocytogenes* uses *Listeria* adhesion protein (LAP) to promote bacterial transepithelial translocation, and induces expression of LAP receptor Hsp60. Infect Immun 78:5062–5073. doi:10.1128/IAI.00516-10. 20876294 PMC2981324

[5] Kim H, Bhunia AK 2013 Secreted *Listeria* adhesion protein (Lap) influences Lap-mediated *Listeria monocytogenes* paracellular translocation through epithelial barrier. Gut Pathog 5:16. doi:10.1186/1757-4749-5-16. 23799938 PMC3716925

[6] Jaradat ZW, Wampler JW, Bhunia AK 2003 A *Listeria* adhesion protein-deficient *Listeria monocytogenes* strain shows reduced adhesion primarily to intestinal cell lines. Med Microbiol Immunol 192:85–91. https://www.ars.usda.gov/research/project/?accnNo=429773. 12736821 10.1007/s00430-002-0150-1

[7] Burkholder KM, Kim KP, Mishra KK, Medina S, Hahm BK, Kim H, Bhunia AK 2009 Expression of LAP, a SecA2-dependent secretory protein, is induced under anaerobic environment. Microbes Infect 11:859–867. doi:10.1016/j.micinf.2009.05.006. 19454322

[8] Orsi RH, den Bakker HC, Wiedmann M 2011 *Listeria monocytogenes* lineages: genomics, evolution, ecology, and phenotypic characteristics. Int J Med Microbiol 301:79–96. doi:10.1016/j.ijmm.2010.05.002. 20708964

[9] Bécavin C, Bouchier C, Lechat P, Archambaud C, Creno S, Gouin E, Wu Z, Kühbacher A, Brisse S, Pucciarelli MG, García-del Portillo F, Hain T, Portnoy DA, Chakraborty T, Lecuit M, Pizarro-Cerdá J, Moszer I, Bierne H, Cossart P 2014 Comparison of widely used *Listeria monocytogenes* strains EGD, 10403S, and EGD-e highlights genomic variations underlying differences in pathogenicity. mBio 5:e00969-14. doi:10.1128/mBio.00969-14. 24667708 PMC3977354

